# (*E*)-4-Chloro-*N*-[(*E*)-2-methyl-3-phenyl­allyl­idene]aniline

**DOI:** 10.1107/S1600536809001871

**Published:** 2009-01-23

**Authors:** Aliakbar D. Khalaji, Jim Simpson

**Affiliations:** a Department of Science, Gorgan University of Agricultural Sciences and Natural Resources, Gorgan, Iran; b Department of Chemistry, University of Otago, PO Box 56, Dunedin, New Zealand

## Abstract

The title Schiff base compound, C_16_H_14_ClN, adopts *E* configurations with respect to both the C=C and C=N bonds. The dihedral angle between the two aromatic rings is 53.27 (4)°, while the plane through the C=C—C=N system is inclined at 9.06 (8)° to the benzene ring and 44.92 (5)° to the chloro­benzene ring. In the crystal structure, weak C—H⋯Cl and C—H⋯N hydrogen bonds stack the mol­ecules down the *a* axis.

## Related literature

For background to the use of Schiff bases as ligands see: Khalaji *et al.* (2008*a* ▶,*b* ▶); and for their bio-activity, see: Karthikeyan *et al.* (2006 ▶); Xiong *et al.* (2008 ▶); Sriram *et al.* (2006 ▶). For related structures, see: Khalaji *et al.* (2007 ▶); Khalaji & Harrison (2008 ▶); Khalaji *et al.* (2008*c* ▶). For reference structural data, see: Allen *et al.* (1987 ▶).
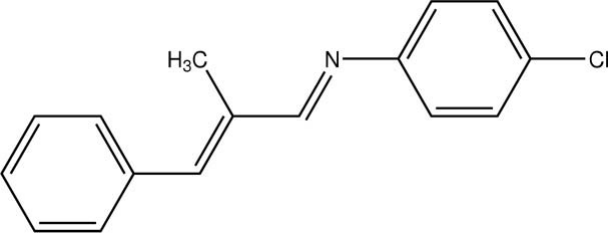


## Experimental

### 

#### Crystal data

C_16_H_14_ClN
*M* *_r_* = 255.73
Orthorhombic, 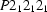

*a* = 7.2486 (10) Å
*b* = 11.6637 (17) Å
*c* = 15.598 (2) Å
*V* = 1318.7 (3) Å^3^
*Z* = 4
Mo *K*α radiation
μ = 0.27 mm^−1^
*T* = 89 (2) K
0.36 × 0.24 × 0.03 mm

#### Data collection

Bruker APEXII CCD area-detector diffractometer
Absorption correction: multi-scan (*SADABS*; Bruker, 2006 ▶) *T*_min_ = 0.841, *T*_max_ = 0.992
21077 measured reflections
4077 independent reflections
3517 reflections with *I* > 2σ(*I*)
*R*_int_ = 0.058

#### Refinement

*R*[*F* ^2^ > 2σ(*F* ^2^)] = 0.039
*wR*(*F* ^2^) = 0.109
*S* = 1.06
4077 reflections
164 parameters
H-atom parameters constrained
Δρ_max_ = 0.30 e Å^−3^
Δρ_min_ = −0.36 e Å^−3^
Absolute structure: Flack (1983 ▶), 1742 Friedel pairs
Flack parameter: 0.01 (6)

### 

Data collection: *APEX2* (Bruker, 2006 ▶); cell refinement: *APEX2* and *SAINT* (Bruker, 2006 ▶); data reduction: *SAINT*; program(s) used to solve structure: *SHELXS97* (Sheldrick, 2008 ▶); program(s) used to refine structure: *SHELXL97* (Sheldrick, 2008 ▶) and *TITAN* (Hunter & Simpson, 1999 ▶); molecular graphics: *SHELXTL* (Sheldrick, 2008 ▶) and *Mercury* (Macrae *et al.*, 2006 ▶); software used to prepare material for publication: *SHELXL97*, *enCIFer* (Allen *et al.*, 2004 ▶), *PLATON* (Spek, 2003 ▶) and *publCIF* (Westrip, 2009 ▶).

## Supplementary Material

Crystal structure: contains datablocks global, I. DOI: 10.1107/S1600536809001871/pk2147sup1.cif

Structure factors: contains datablocks I. DOI: 10.1107/S1600536809001871/pk2147Isup2.hkl

Additional supplementary materials:  crystallographic information; 3D view; checkCIF report

## Figures and Tables

**Table 1:** Hydrogen-bond geometry (Å, °)

*D*—H⋯*A*	*D*—H	H⋯*A*	*D*⋯*A*	*D*—H⋯*A*
C7—H7⋯N1^i^	0.95	2.67	3.524 (2)	150
C13—H13⋯Cl1^ii^	0.95	2.92	3.7311 (17)	144

Symmetry codes: (i) 
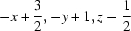
; (ii) 
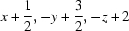
.

**Fig. 1.**
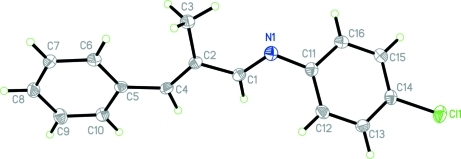
The structure of (I) with displacement ellipsoids for the non-hydrogen atoms drawn at the 50% probability level.

**Fig. 2.**
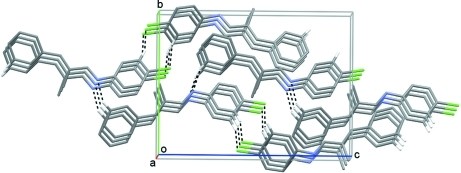
Crystal packing of (I) viewed down the a axis with hydrogen bonds drawn as dashed lines. H atoms not involved in hydrogen bonding have been omitted.

## supplementary crystallographic information

### Comment

Schiff-bases are well known chelating ligands in coordination chemistry (Khalaji *et al.*, 2008*a*,b), and exhibit a wide range of biological activities (Karthikeyan *et al.*, 2006) including anti-HIV activity (Xiong *et al.*, 2008; Sriram *et al.*, 2006). As a continuation of our work on the synthesis and structural characterization of Schiff-base compounds (Khalaji *et al.*, 2007; Khalaji & Harrison, 2008; Khalaji *et al.*, 2008*c*), we report here the structure of the title compound, C_16_H_14_NCl, (I), Fig 1.

The title Schiff-base compound, C_16_H_14_NCl, adopts *E* configurations with respect to both the C2=C4 and C1=N1 bonds. Bond lengths in the molecule are normal (Allen, *et al.*, 1987) and similar to those found in related compounds (Khalaji *et al.*, 2007; Khalaji & Harrison, 2008; Khalaji *et al.*, 2008*c*). The dihedral angle between the two aromatic rings is 53.27 (4)° while the plane through the C2=C4–C1=N1 system is inclined at 9.06 (8)° to the C5···C10 ring and 44.92 (5)° to the C11···C16 ring.

In the crystal structure, weak C13—H13···Cl1 and C7—H7···N1 hydrogen bonds stack the molecules down the *a* axis.

### Experimental

The title compound was prepared in 76% yield from 4-chloroaniline and α-methylcinnamaldehyde as reported elsewhere (Khalaji *et al.* 2007) and recrystallized from methanol.

### Refinement

The H atom bound to N1 was located in a difference electron density map and refined freely with an isotropic displacement parameter. All other H-atoms were refined using a riding model with d(C—H) = 0.95 Å, *U*_iso_= 1.2*U*_eq_ (C) for aromatic and 0.98 Å, *U*_iso_ = 1.5*U*_eq_ (C) for CH_3_ H atoms.

### Crystal data

**Table:**

C_16_H_14_ClN	*F*(000) = 536
*M**_r_* = 255.73	*D*_x_ = 1.288 Mg m^−^^3^
Orthorhombic, *P*2_1_2_1_2_1_	Mo *K*α radiation, λ = 0.71073 Å
Hall symbol: P 2ac 2ab	Cell parameters from 5396 reflections
*a* = 7.2486 (10) Å	θ = 2.6–28.8°
*b* = 11.6637 (17) Å	µ = 0.27 mm^−^^1^
*c* = 15.598 (2) Å	*T* = 89 K
*V* = 1318.7 (3) Å^3^	Rectangular plate, pale yellow
*Z* = 4	0.36 × 0.24 × 0.03 mm

### Data collection

**Table:**

Bruker APEXII CCD area-detector diffractometer	4077 independent reflections
Radiation source: fine-focus sealed tube	3517 reflections with *I* > 2σ(*I*)
graphite	*R*_int_ = 0.058
ω scans	θ_max_ = 30.7°, θ_min_ = 3.1°
Absorption correction: multi-scan (*SADABS*; Bruker, 2006)	*h* = −10→10
*T*_min_ = 0.841, *T*_max_ = 0.992	*k* = −16→16
21077 measured reflections	*l* = −21→22

### Refinement

**Table:**

Refinement on *F*^2^	Secondary atom site location: difference Fourier map
Least-squares matrix: full	Hydrogen site location: inferred from neighbouring sites
*R*[*F*^2^ > 2σ(*F*^2^)] = 0.039	H-atom parameters constrained
*wR*(*F*^2^) = 0.109	*w* = 1/[σ^2^(*F*_o_^2^) + (0.0649*P*)^2^] where *P* = (*F*_o_^2^ + 2*F*_c_^2^)/3
*S* = 1.06	(Δ/σ)_max_ = 0.001
4077 reflections	Δρ_max_ = 0.30 e Å^−^^3^
164 parameters	Δρ_min_ = −0.36 e Å^−^^3^
0 restraints	Absolute structure: Flack (1983), 1742 Friedel pairs
Primary atom site location: structure-invariant direct methods	Flack parameter: 0.01 (6)

### Special details

**Table:**

Geometry. All e.s.d.'s (except the e.s.d. in the dihedral angle between two l.s. planes) are estimated using the full covariance matrix. The cell e.s.d.'s are taken into account individually in the estimation of e.s.d.'s in distances, angles and torsion angles; correlations between e.s.d.'s in cell parameters are only used when they are defined by crystal symmetry. An approximate (isotropic) treatment of cell e.s.d.'s is used for estimating e.s.d.'s involving l.s. planes.
Refinement. Refinement of *F*^2^ against ALL reflections. The weighted *R*-factor *wR* and goodness of fit *S* are based on *F*^2^, conventional *R*-factors *R* are based on *F*, with *F* set to zero for negative *F*^2^. The threshold expression of *F*^2^ > σ(*F*^2^) is used only for calculating *R*-factors(gt) *etc*. and is not relevant to the choice of reflections for refinement. *R*-factors based on *F*^2^ are statistically about twice as large as those based on *F*, and *R*- factors based on ALL data will be even larger.

### Fractional atomic coordinates and isotropic or equivalent isotropic displacement parameters (Å^2^)

**Table:**

	*x*	*y*	*z*	*U*_iso_*/*U*_eq_	
N1	0.5152 (2)	0.52967 (12)	0.68870 (9)	0.0202 (3)	
C1	0.4972 (2)	0.61272 (13)	0.63497 (9)	0.0183 (3)	
H1	0.4550	0.6848	0.6553	0.022*	
C2	0.5394 (2)	0.60026 (13)	0.54384 (9)	0.0172 (3)	
C3	0.6040 (3)	0.48397 (13)	0.51348 (10)	0.0224 (3)	
H3A	0.5303	0.4601	0.4639	0.034*	
H3B	0.5892	0.4280	0.5598	0.034*	
H3C	0.7343	0.4884	0.4970	0.034*	
C4	0.5161 (2)	0.69557 (13)	0.49535 (10)	0.0178 (3)	
H4	0.4725	0.7602	0.5264	0.021*	
C5	0.5458 (2)	0.71712 (13)	0.40389 (10)	0.0166 (3)	
C6	0.6332 (3)	0.64156 (14)	0.34605 (10)	0.0227 (3)	
H6	0.6771	0.5695	0.3659	0.027*	
C7	0.6560 (2)	0.67119 (14)	0.26031 (10)	0.0228 (3)	
H7	0.7151	0.6192	0.2223	0.027*	
C8	0.5931 (2)	0.77606 (14)	0.22974 (10)	0.0228 (3)	
H8	0.6072	0.7953	0.1709	0.027*	
C9	0.5089 (3)	0.85283 (15)	0.28615 (11)	0.0243 (4)	
H9	0.4672	0.9252	0.2660	0.029*	
C10	0.4861 (2)	0.82324 (14)	0.37165 (10)	0.0205 (3)	
H10	0.4286	0.8762	0.4094	0.025*	
C11	0.4819 (2)	0.55276 (13)	0.77639 (10)	0.0182 (3)	
C12	0.5526 (2)	0.64996 (13)	0.81773 (10)	0.0203 (3)	
H12	0.6233	0.7040	0.7862	0.024*	
C13	0.5200 (2)	0.66792 (13)	0.90454 (10)	0.0207 (3)	
H13	0.5671	0.7342	0.9323	0.025*	
C14	0.4183 (2)	0.58807 (13)	0.94995 (10)	0.0193 (3)	
Cl1	0.38085 (6)	0.60910 (4)	1.05938 (2)	0.02595 (11)	
C15	0.3494 (2)	0.48964 (13)	0.91081 (11)	0.0206 (3)	
H15	0.2800	0.4354	0.9428	0.025*	
C16	0.3841 (2)	0.47222 (13)	0.82400 (10)	0.0199 (3)	
H16	0.3404	0.4045	0.7969	0.024*	

### Atomic displacement parameters (Å^2^)

**Table:**

	*U*^11^	*U*^22^	*U*^33^	*U*^12^	*U*^13^	*U*^23^
N1	0.0204 (7)	0.0212 (6)	0.0190 (6)	0.0000 (5)	0.0007 (5)	0.0002 (5)
C1	0.0174 (7)	0.0186 (6)	0.0189 (7)	0.0011 (6)	0.0001 (6)	−0.0018 (6)
C2	0.0162 (7)	0.0171 (6)	0.0182 (7)	0.0014 (6)	−0.0009 (5)	−0.0021 (6)
C3	0.0278 (9)	0.0173 (6)	0.0222 (8)	0.0029 (7)	0.0032 (7)	−0.0004 (6)
C4	0.0186 (8)	0.0163 (7)	0.0185 (7)	0.0000 (6)	0.0011 (6)	−0.0038 (5)
C5	0.0145 (7)	0.0161 (6)	0.0191 (7)	−0.0025 (6)	0.0003 (6)	−0.0018 (5)
C6	0.0291 (9)	0.0164 (6)	0.0225 (8)	0.0000 (7)	0.0035 (7)	−0.0014 (5)
C7	0.0280 (9)	0.0206 (7)	0.0199 (7)	−0.0031 (7)	0.0053 (6)	−0.0036 (6)
C8	0.0244 (9)	0.0264 (7)	0.0175 (7)	−0.0045 (7)	−0.0010 (6)	0.0008 (6)
C9	0.0257 (9)	0.0238 (7)	0.0233 (8)	0.0035 (7)	0.0006 (7)	0.0054 (6)
C10	0.0204 (8)	0.0198 (7)	0.0213 (7)	0.0028 (6)	0.0019 (6)	0.0004 (6)
C11	0.0174 (7)	0.0190 (7)	0.0183 (7)	0.0034 (6)	−0.0007 (6)	0.0014 (6)
C12	0.0209 (8)	0.0183 (6)	0.0218 (7)	−0.0003 (6)	−0.0028 (6)	0.0042 (6)
C13	0.0233 (8)	0.0174 (7)	0.0214 (7)	−0.0008 (6)	−0.0039 (6)	−0.0012 (6)
C14	0.0181 (7)	0.0217 (7)	0.0180 (7)	0.0051 (6)	−0.0004 (6)	0.0021 (6)
Cl1	0.0302 (2)	0.02978 (19)	0.01791 (17)	0.00604 (18)	0.00123 (15)	−0.00069 (15)
C15	0.0197 (8)	0.0196 (7)	0.0225 (7)	0.0009 (6)	0.0016 (6)	0.0030 (6)
C16	0.0199 (8)	0.0172 (6)	0.0228 (7)	0.0000 (6)	−0.0005 (6)	−0.0006 (6)

### Geometric parameters (Å, °)

**Table:**

N1—C1	1.287 (2)	C8—C9	1.396 (2)
N1—C11	1.415 (2)	C8—H8	0.9500
C1—C2	1.4612 (19)	C9—C10	1.387 (2)
C1—H1	0.9500	C9—H9	0.9500
C2—C4	1.355 (2)	C10—H10	0.9500
C2—C3	1.511 (2)	C11—C16	1.392 (2)
C3—H3A	0.9800	C11—C12	1.401 (2)
C3—H3B	0.9800	C12—C13	1.390 (2)
C3—H3C	0.9800	C12—H12	0.9500
C4—C5	1.464 (2)	C13—C14	1.383 (2)
C4—H4	0.9500	C13—H13	0.9500
C5—C10	1.404 (2)	C14—C15	1.393 (2)
C5—C6	1.412 (2)	C14—Cl1	1.7456 (16)
C6—C7	1.391 (2)	C15—C16	1.392 (2)
C6—H6	0.9500	C15—H15	0.9500
C7—C8	1.390 (2)	C16—H16	0.9500
C7—H7	0.9500		
			
C1—N1—C11	117.96 (14)	C7—C8—H8	120.3
N1—C1—C2	122.50 (14)	C9—C8—H8	120.3
N1—C1—H1	118.8	C10—C9—C8	119.89 (15)
C2—C1—H1	118.8	C10—C9—H9	120.1
C4—C2—C1	115.80 (14)	C8—C9—H9	120.1
C4—C2—C3	126.88 (13)	C9—C10—C5	121.79 (15)
C1—C2—C3	117.32 (13)	C9—C10—H10	119.1
C2—C3—H3A	109.5	C5—C10—H10	119.1
C2—C3—H3B	109.5	C16—C11—C12	119.14 (15)
H3A—C3—H3B	109.5	C16—C11—N1	118.32 (14)
C2—C3—H3C	109.5	C12—C11—N1	122.44 (15)
H3A—C3—H3C	109.5	C13—C12—C11	120.52 (15)
H3B—C3—H3C	109.5	C13—C12—H12	119.7
C2—C4—C5	131.77 (14)	C11—C12—H12	119.7
C2—C4—H4	114.1	C14—C13—C12	119.20 (15)
C5—C4—H4	114.1	C14—C13—H13	120.4
C10—C5—C6	117.38 (14)	C12—C13—H13	120.4
C10—C5—C4	117.05 (14)	C13—C14—C15	121.45 (15)
C6—C5—C4	125.55 (14)	C13—C14—Cl1	119.28 (12)
C7—C6—C5	120.84 (15)	C15—C14—Cl1	119.25 (12)
C7—C6—H6	119.6	C16—C15—C14	118.82 (15)
C5—C6—H6	119.6	C16—C15—H15	120.6
C8—C7—C6	120.63 (15)	C14—C15—H15	120.6
C8—C7—H7	119.7	C11—C16—C15	120.83 (14)
C6—C7—H7	119.7	C11—C16—H16	119.6
C7—C8—C9	119.46 (15)	C15—C16—H16	119.6

### Hydrogen-bond geometry (Å, °)

**Table:**

*D*—H···*A*	*D*—H	H···*A*	*D*···*A*	*D*—H···*A*
C7—H7···N1^i^	0.95	2.67	3.524 (2)	150
C13—H13···Cl1^ii^	0.95	2.92	3.7311 (17)	144

Symmetry codes: (i) −*x*+3/2, −*y*+1, *z*−1/2; (ii) *x*+1/2, −*y*+3/2, −*z*+2.
